# Spatiotemporal patterns and driving mechanisms of provincial road traffic accidents in China, 2016–2024

**DOI:** 10.3389/fpubh.2026.1914301

**Published:** 2026-08-27

**Authors:** Jun Zhu, Rui Zheng, Zhizhuang Duan

**Affiliations:** Xingzhi College, Zhejiang Normal University, Lanxi, China

**Keywords:** driving mechanisms, geographical detector, spatial heterogeneity, traffic accidents, traffic safety

## Abstract

**Introduction:**

Road traffic accidents have become a major public health issue of global concern.

**Methods:**

Drawing on panel data from 31 provincial-level regions in China from 2016 to 2024, this study develops an evaluation framework comprising ten indicators related to transport infrastructure, socioeconomic development, and the natural environment. It employs the imbalance index, geographical concentration index, standard deviational ellipse, spatial autocorrelation analysis, and the geographical detector model to examine the spatiotemporal evolution and multidimensional driving mechanisms of provincial traffic accidents in China.

**Results:**

The results show substantial interprovincial imbalance but weak overall spatial adjacency. Global Moran's I does not indicate significant spatial correlation, while the distribution center shifted generally southwestward and the standard deviational ellipse expanded and then contracted. The geographical detector results indicate that the number of motor vehicle drivers, resident population density, gross regional product, and civilian vehicle ownership are the main factors explaining spatial differentiation. Temperature, precipitation, and total road mileage have moderate individual explanatory power, while higher educational-attainment strata are associated with lower mean accident counts. Most factor pairs exhibit bivariate or nonlinear enhancement.

**Discussion:**

These findings suggest that traffic accidents are shaped by the multidimensional coupling of “people-vehicles-roads-environment” and provide evidence for regionally differentiated traffic-safety policies.

## 1 Introduction

Traffic accidents have become a major global public health and social problem. According to the World Health Organization (WHO), approximately 1.19 million people die each year in road traffic crashes worldwide (1). China, as one of the world's most populous and fastest-growing developing countries, faces serious challenges in road traffic safety (2, 3). Recent estimates based on Global Burden of Disease 2021 data indicate that China's age-standardized road traffic mortality rate was 4.38 deaths per 100,000 population, lower than the global average of 5.96, but road traffic injuries continue to impose a considerable burden because of the country's large population, rapid motorization, and regional disparities (4). National statistics also show that casualty-involving traffic accidents increased from 212,846 to 260,072 between 2016–2024. These developments underscore the need to examine the spatiotemporal evolution and driving mechanisms of road traffic accidents from a macro-level perspective.

Existing studies have extensively examined the causes and spatiotemporal distribution of traffic accidents. Scholars in different countries have analyzed the accident heterogeneity in relation to local geographical environments and socioeconomic conditions. GIS-based techniques, including spatial autocorrelation and kernel density estimation), haverevealed how urban expansion and road network configuration are associated with the evolution of traffic-accident hotspots (5, 6). Long-term regional studies further show that geographical constraints and environmental changes are associated with the formation of accident clusters (7). These studies establish the importance of spatial scale, local context, and multiple interacting conditions.

Although these studies have advanced understanding of the spatiotemporal characteristics of traffic accidents, findings from specific national or regional contexts cannot be directly generalized to China's heterogeneous transportation system. Regional differences in infrastructure investment road capacity, motorization and population exposure create distinct provincial conditions (8). Chinese studies have identified temporally and specially constrained associations between driver violations and accident severity (9). Research on bicycles and electric bicycles in Guangzhou has also documented the distinctive casualty patterns among vulnerable road users (10). However, most studies focus on single-city cross-sections or specific road segments, and continuous tracking of long-term national patterns remains limited. Conventional linear regression and spatial econometric models may also difficult to interpret under multicollinearity and may overlook nonlinear effects generated by the multiple interacting factors. A nationwide framework therefore needed to capture long-term spatiotemporal evolution and interactions among transport, socioeconomic, demographic, and environmental factors.

To address these gaps, this study draws on panel data from 31 provincial-level regions in China (excluding Hong Kong, Macao, and Taiwan) from 2016 to 2024 and develops an integrated framework incorporating transport infrastructure, socioeconomic development, demographic characteristics, and environmental conditions. The geographical model is a spatial statistical approach that does not rely on the assumptions of conventional linear regression. Rather than estimating regression coefficients, it quantifies the explanatory power of individual factors through spatially stratified heterogeneity and evaluates whether paired factors enhance or weaken their explanatory power. This feature reduces the influence of multicollinearity and supports examination of the complex-nonlinear relationships. This study therefore provides a nationwide perspective on provincial accident evolution and examines reveals their multidimensional driving mechanisms within the people–vehicles–roads–environment framework, offering evidence for region-specific traffic-safety policies and risk-prevention strategies.

## 2 Literature review

### 2.1 Transport facilities and system exposure

Transport infrastructure constitutes the physical setting for regional mobility, and its scale and structure shape macro-level traffic exposure. Total road mileage and civilian vehicle ownership are widely used as proxy indicators of infrastructure and motorization. Although neither directly represents transport supply or travel demand, together they capture dimensions of the transportation system associated with accident risk (11, 12).

Road network characteristics are associated with the spatial distribution of traffic accidents. But this relationship should be interpreted together with road design, traffic composition, and local driving conditions (13). In China, road mileage should be understood as an indicator of infrastructure scale rather than a direct determinant of accident occurrence (14). Large-scale road construction in central and western China has expanded regional road networks and connectivity. Together with increasing freight mobility, this expansion has increased exposure in some corridors. Mountainous corridors may remain vulnerable where supporting facilities and management do not keep pace with network expansion (15).

Growth in civilian vehicle ownership reflects increasing motorization and potential traffic exposure. When accompanied by intensive traffic activity and limited management capacity, higher motorization may increase traffic conflicts and accident clustering. Rapid ownership can increase rear-end collisions, sideswipe crashes, and other conflicts under complex operating conditions (16). When vehicle growth exceeds the carrying and upgrading capacity of infrastructure, high-density road network areas may more likely to become accident hot spots (17). Evidence from Jinan likewise suggests a positive spatial association between civilian vehicle ownership and traffic accidents (18).

Research on traffic exposure nevertheless remains concentrated on single cities or specific road networks. Recent studies integrating built-environment characteristics with dynamic traffic-flow variables emphasize that exposure is a dynamic process rather than a static infrastructure condition (19). Although geographically weighted regression, it is limited in addressing multicollinearity and nonlinear interaction effects. This restricts the generalizability of existing local findings to broader macro-regional scales and leaves the combined role of infrastructure and exposure insufficiently understood.

### 2.2 Population and socioeconomic dimensions

Population structure, educational attainment, and economic development traffic risk by influencing travel demand, risk perception and safety capacity.

Urbanization and resident population density influence both the volume and form of mobility. Highly urbanized and dense may experience severe congestion, which can reduce average speeds and the likelihood of fatal crashes even when property-damage accidents remain frequent (20). By contrast, research on urban–rural differences in China higher accident fatality rates in some rural areas with lower density and urbanization (21). Points of interest and population mobility in built-up also contribute spatiotemporal variation in accident distribution (22). These contrasting patterns indicate that the relationship between population concentration and accident risk may be nonlinear and dependent on development stage.

Educational attainment is an important human dimension of traffic safety. Drivers with higher education may show stronger compliance with traffic regulations and a greater ability to respond to complex road conditions (23). Urban–rural differences in violations and driver composition further suggest that human factors contribute to the spatial distribution and severity of accidents (21). At the regional level, improved educational attainment may also contribute to a more orderly traffic environment and fewer serious accidents associated with violations or fatigue driving (24).

Gross regional product and disposable income reflect travel frequency and vehicle purchasing power as well as investment in road, enforcement, vehicle safety and emergency services (25). Their net association with accidents may therefore vary across development stages. Previous evidence suggests an inverted U-shaped relationship between economic development and road traffic economic development and injuries (27). County-level research likewise identifies spatially heterogeneous and nonlinear effects of population composition, education, commuting, and infrastructure accessibility on overall and fatal crash rates (28).

Overall, socioeconomic effects on traffic accidents are staged, nonlinear, and sensitive to research scales, supporting systematic examination from a national macro-level perspective.

### 2.3 Dynamic disturbance effects of the natural environment

Precipitation can destabilize the traffic system by reducing visibility pavement fiction, and vehicle control. Its spatial distribution may also overlaps with regional accident-prone areas (5). Machine-learning analyses indicate that heavy precipitation combined with poor road conditions can substantially increases accident risk (29). Evidence from Shenzhen identifies a positive association between hourly precipitation and road traffic casualties, particularly during period of frequent summer rainfall (2). Temperature-related risks should also be considered. Temperature fluctuations may affect driver fatigue, emotional stress, tires, vehicle components, and road surfaces. Research on spatiotemporal instability further indicates that extreme temperatures and associated weather conditions may worsen visibility and increase vehicle failures, although their implications vary across traffic and infrastructure settings (30).

Research on natural-environment effects of has shifted from single factors analysis toward multi-factor coupling, but meteorological variables are still often considered separately from traffic exposure and socioeconomic conditions. This limits assessment of their joint role within the “people–vehicles–roads–environment” system.

## 3 Methodology

### 3.1 Data sources and indicator system

This study examines the spatiotemporal distribution and driving mechanisms of traffic accidents across 31 provincial-level regions in China, from 2016 to 2024. Hong Kong, Macao, and Taiwan are excluded because data availability and statistical consistency. The spatial differentiation of traffic accidents reflects the combined conditions of people, vehicles, roads and the environment. Ten indicators represent this system: total road mileage, urbanization rate, gross regional product, number of motor vehicle drivers, civilian vehicle ownership, disposable income, resident population density, precipitation, temperature, and educational attainment. Temperature and precipitation were obtained from the National Tibetan Plateau Data Center of China (https://data.tpdc.ac.cn/home), administrative boundaries from the National Geomatics Center of China (https://www.ngcc.cn/), and the remaining data from the official website of the National Bureau of Statistics of China (https://www.stats.gov.cn/). Because population density based on the 2020 census and sample surveys in other years, missing observations were estimated by trend fitting anchored to the census values.

For geographical detector analysis, all independent variables were discretized using the natural breaks method in ArcGIS 10.8.1. Classification used pooled observations from all provinces and years (31 provinces × 9 years), rather than separate annual classifications. This unified strategy keeps stratum threshold consistent over time and improves comparability. Each indicator was divided into five strata (Table 1). The number of strata was selected to preserve differences among levels while retaining adequate observations within each group. Because provincial variables differed substantially in scale and several were skewed, natural breaks was used to minimize within-class variance and maximize between-class differences (47, 48).

**Table 1:** Indicator system for influencing factors.

Influencing indicator	Code	Number of strata
Total road mileage	*X*_1_	5
Urbanization rate	*X*_2_	5
Gross regional product	*X*_3_	5
Number of motor vehicle drivers	*X*_4_	5
Number of civilian vehicles	*X*_5_	5
Per capita disposable income	*X*_6_	5
Resident population density	*X*_7_	5
Precipitation	*X*_8_	5
Temperature	*X*_9_	5
Educational attainment	*X*_10_	5

### 3.2 Analytical methods

**Figure 1:**
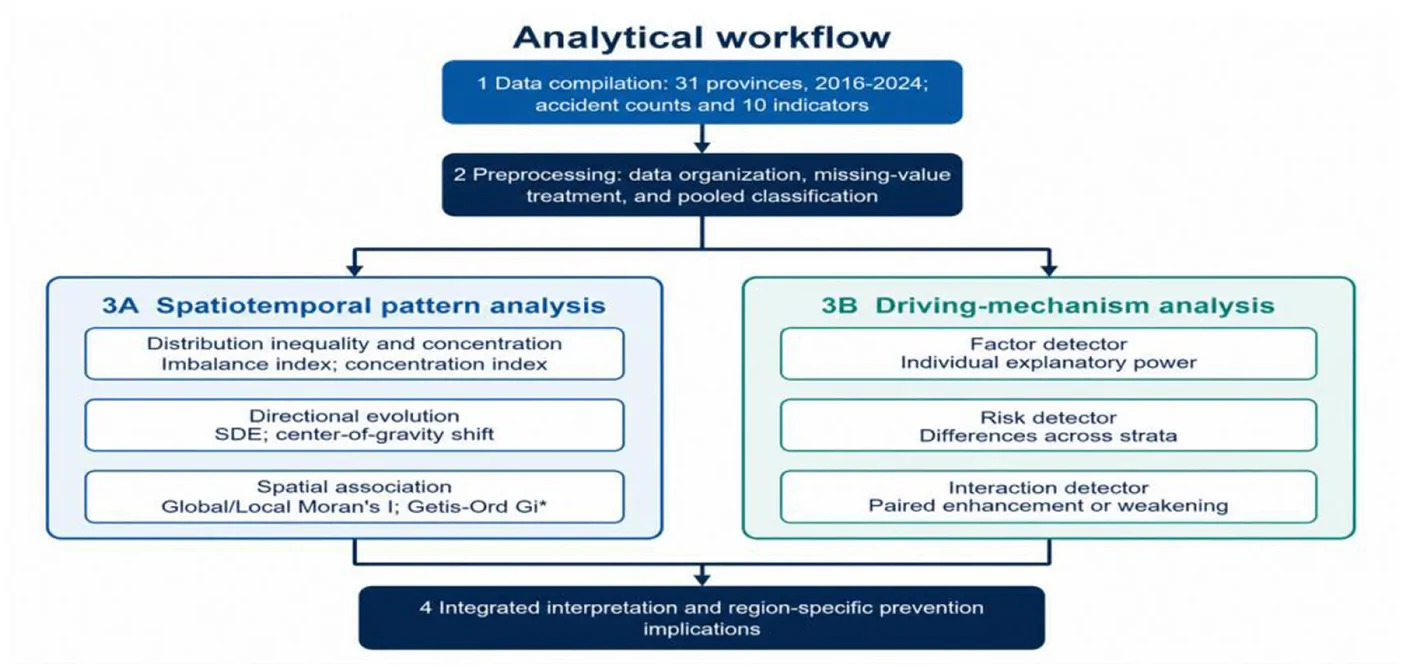
Analytical workflow of the study.

Figure 1 summarizes the analytical workflow. Spatial statistical methods first characterize accident distribution and evolution; the geographical detector then evaluates the explanatory power and interactions effects of the ten indicators.

#### 3.2.1 Imbalance index

The imbalance index evaluates the overall spatial inequality of traffic-accident distribution (31). It ranges from 0 to 1, a larger value indicates that a smaller number of provinces account for a greater share of the national total:


$$\begin{array}{c} S=\frac{\sum_{i=1}^{n}Y_{i}-50\left(n+1\right)}{100n-50\left(n+1\right)} \end{array}$$ (1)

where *n* is the number of provinces and *Y*_*i*_ is the cumulative percentage of accidents from the first to the *i*th province after provincial shares of national traffic accidents are ranked in descending order.

#### 3.2.2 Geographical concentration index (G)

The geographical concentration index measures the degree to which traffic accidents are concentrated in a subset of provinces. A higher value indicates a stronger tendency toward regional concentration


$$\begin{array}{c} G=100\times \sqrt{\overset{n}{\underset{i=1}{\sum }}{\left(\frac{X_{i}}{T}\right)}^{2}} \end{array}$$ (2)

#### 3.2.3 Standard deviational ellipse (SDE) and center of gravity shift

The standard deviational ellipse and center-of-gravity analysis describe directional evolution and migration. The center of gravity records the accident-weighted mean location of provincial centroids for each year:


$$\begin{array}{c} X=\frac{\sum_{i=1}^{n}w_{i}x_{i}}{\sum_{i=1}^{n}w_{i}},\;Y=\frac{\sum_{i=1}^{n}w_{i}y_{i}}{\sum_{i=1}^{n}w_{i}} \end{array}$$ (3)

where (*x*_*i*_, *y*_*i*_) is the centroid of province *i, w*_*i*_ is its accident count, and n is the number of provinces.

The standard deviational ellipse (SDE) summarizes the dispersion and directional characteristics of geographical elements (32). Its major axis describes dispersion in the dominant direction, the minor axis describes dispersion in the secondary direction, and the azimuth records orientation:


$$\begin{array}{c} \sigma_{x}=\sqrt{\frac{\sum_{i=1}^{n}{\left({\tilde{x}}_{i}\cos\theta -{ỹ}_{i}\sin\theta \right)}^{2}}{n}},\\ \sigma_{y}=\sqrt{\frac{\sum_{i=1}^{n}{\left({\tilde{x}}_{i}\sin\theta +{ỹ}_{i}\cos\theta \right)}^{2}}{n}} \end{array}$$ (4)

The azimuth (θ) is the clockwise angle from north to the major axis and is calculated as:


$$\begin{array}{c} \tan\theta =\frac{\left(\sum {\tilde{x}}_{i}^{2}-\sum {ỹ}_{i}^{2}\right)+\sqrt{{\left(\sum {\tilde{x}}_{i}^{2}-\sum {ỹ}_{i}^{2}\right)}^{2}+4{\left(\sum {\tilde{x}}_{i}{ỹ}_{i}\right)}^{2}}}{2\sum {\tilde{x}}_{i}{ỹ}_{i}} \end{array}$$ (5)

where ${\tilde{x}}_{i}$ and ỹ_*i*_ are the deviations of provincial centroid coordinates from the global center of gravity (X, Y).

.

#### 3.2.4 Global Moran's I

Global Moran's I tests whether provincial accidents counts exhibit overall spatial dependence (33):


$$\begin{array}{c} I=\frac{N\sum_{i}\sum_{j}w_{ij}\left(X_{i}-\bar{X}\right)\left(X_{j}-\bar{X}\right)}{\sum_{i}\sum_{j}w_{ij}\sum_{i}{\left(X_{i}-\bar{X}\right)}^{2}} \end{array}$$ (6)

where *X*_*i*_ and *X*_*j*_ are accident counts in provinces *i* and *j*, respectively, $\bar{X}$ is their mean, *w*_*ij*_ is the spatial-weight matrix, and N is the number of provinces. Positive and negative value indicate positive and negative spatial association, respectively. Significance is evaluated jointly using the z-score and *p*-value.

#### 3.2.5 Local indicators of spatial association (LISA)

Local Moran's I, or Local Indicators of Spatial Association (LISA), overcomes the limitation of the global statistic by identifying province-level spatial associations (34):


$$\begin{array}{c} I_{i}=\frac{X_{i}-\bar{X}}{S_{i}^{2}}\overset{N}{\underset{j=1,j\neq i}{\sum }}w_{ij}\left(X_{j}-\bar{X}\right) \end{array}$$ (7)

Based on the significance test, observations are classified as high–high clusters, low–low clusters, high–low outliers, or low–high outliers.

#### 3.2.6 Hot spot analysis: Getis–Ord Gi^*^

Getis–Ord Gi^*^ further measures the statistical intensity of high- and low-value clusters among neighboring provinces and identifies hot and cold spots:


$$\begin{array}{c} G_{i}*=\frac{\sum_{j=1}^{n}w_{ij}X_{j}-\bar{X}\sum_{j=1}^{n}w_{ij}}{S\sqrt{\frac{\left[n\sum_{j=1}^{n}w_{ij}^{2}-{\left(\sum_{j=1}^{n}w_{ij}\right)}^{2}\right]}{n-1}}} \end{array}$$ (8)

Positive z-score above 1.65, 1.96, and 2.58 indicate hot spots at the 90%, 95%, and 99% confidence levels; the corresponding negative values indicate cold spots.

#### 3.2.7 Geodetector model

The geographical detector qualifies spatially stratified heterogeneity without assuming linear functional form (35). The four model includes factor, interaction, risk, and ecological detectors. This study uses the factor detector to measure individual explanatory power, the interaction detector to compare paired effects, and the risk detector to compare mean accident counts across strata. The factor-detector q statistic is:


$$\begin{array}{c} q=1-\frac{\sum_{h=1}^{L}N_{h}\sigma_{h}^{2}}{N\sigma^{2}}=1-\frac{SSW}{SST} \end{array}$$ (9)

Where *N*_*h*_ and $\sigma_{h}^{2}$ are the sample size and variance within stratum h, and *N* and σ^2^ are their counterparts for the full study area. The *q* statistic ranges from 0 to 1, with larger values indicating greater explanatory power.

The interaction detector compares q (X1∩X2) with the individual q values to determine whether their joint explanatory power is weakened, independent, enhanced, or nonlinearly enhanced (Table 2).

**Table 2:** Criteria for judging interaction type.

Basis for judgment	Type of interaction
*q*(*X*_1_ ∩ *X*_2_) < *Min*[*q*(*X*_1_), *q*(*X*_2_)]	Nonlinear weakening
*Min*[*q*(*X*_1_), *q*(*X*_2_)] < *q*(*X*_1_ ∩ *X*_2_) < *Max*[*q*(*X*_1_), *q*(*X*_2_)]	Univariate weakening
*q*(*X*_1_ ∩ *X*_2_) > *Max*[*q*(*X*_1_), *q*(*X*_2_)]	Bi-factor enhancement
*q*(*X*_1_ ∩ *X*_2_) = *q*(*X*_1_) + *q*(*X*_2_)	Mutually independent
*q*(*X*_1_ ∩ *X*_2_) > *q*(*X*_1_) + *q*(*X*_2_)	Nonlinear enhancement

The risk detector determines whether mean accident counts differ significantly across geographical subregions corresponding to different strata. Difference between two strata are tested using:


$$\begin{array}{c} t_{{\overset{¯}{y}}_{h=1}-{\overset{¯}{y}}_{h=2}}=\frac{{\overset{¯}{Y}}_{h=1}-{\overset{¯}{Y}}_{h=2}}{\sqrt{\frac{\sigma_{h=1}^{2}}{N_{h=1}}+\frac{\sigma_{h=2}^{2}}{N_{h=2}}}} \end{array}$$ (10)

where ${\overset{¯}{Y}}_{h}$, *N*_*h*_, and $\sigma_{h}^{2}$ are the mean accident count, sample size, and variance of stratum. The resulting comparisons describe differences between strata rather than individual-level risk thresholds.

## 4 Results

Result are presented first for provincial spatiotemporal patterns and then for the geographical detector analysis within the people–vehicles–roads–environment framework.

### 4.1 Spatiotemporal distribution characteristics

The imbalance and concentration indices show persistent spatial unevenness and phased fluctuations (Table 3).

**Table 3:** Imbalance index and geographical concentration index of traffic accidents in China, 2016–2024.

Year	Imbalance index (S)	Geographical concentration index (G)
2016	0.4159	22.81
2017	0.4020	22.59
2018	0.4105	22.32
2019	0.4259	22.67
2020	0.4145	22.56
2021	0.4323	23.16
2022	0.4466	23.66
2023	0.4480	24.33
2024	0.4239	23.99

The imbalance index ranged from 0.4020 to 0.4480, showing a fluctuating increase, followed by slight decline. It declined temporarily in 2020, increased from 2021 to 2023, and eased in 2024. The concentration index remained above the equal-distribution benchmark of 17.96 and ranged from 22.32 to 24.33. Traffic accident were therefore unevenly distributed across provinces, with a limited group accounting for relatively large shares. However, the values did not indicate a high degree of concentration, and the national pattern remained broadly dispersed.

**Figure 2:**
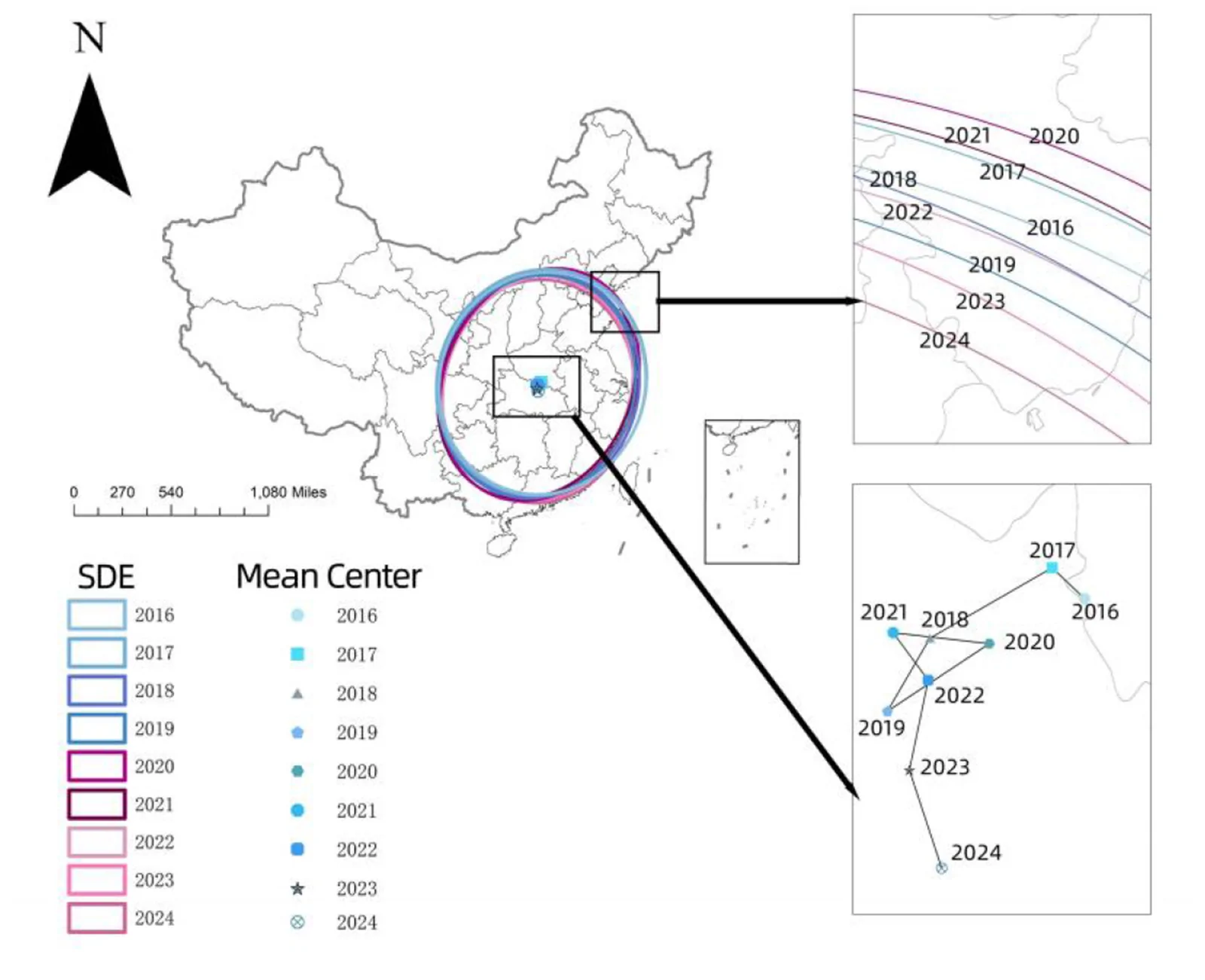
SDE and center-of-gravity shift of traffic accidents in China, 2016–2024.

The SDE results show expansion followed by contraction, while the center of gravity shifted generally southwestward (Table 4, Figure 2). CenterX declined from 347,657.6859 in 2016 to 310,225.7812 in 2024, and CenterY from 3,415,579.402 to 3,344,944.364, with a clearer downward tendency after 2021. YStdDist exceeded XStdDist in every year, indicating dominant north–south dispersion. XStdDist declined through 2022 before rising slightly, indicating an overall east–west contraction followed by limited expansion. YStdDist increased and then returned close to its 2016 level. The rotation angle also changed substantially, especially after 2021. Although the annual centers appear close on the map, these parameters quantify changes in location, dispersion, and orientation that are difficult to distinguish visually.

**Table 4:** SDE and center of gravity parameters of traffic accidents in China, 2016–2024.

Year	CenterX	CenterY	XStdDist	YStdDist	Rotation
2016	347,657.6859	3,415,579.402	914,737.9902	996,879.0318	16.070964
2017	339,134.6726	3,423,661.76	927,825.8194	1,018,962.556	18.55799
2018	307,154.8931	3,405,212.845	900,875.3511	1,012,105.344	11.314383
2019	296,006.3891	3,386,092.554	868,969.0665	1,005,790.924	16.912035
2020	322,684.3629	3,403,779.345	851,076.8687	1,069,320.775	23.386284
2021	297,505.9277	3,406,657.835	844,579.1012	1,056,265.018	24.325663
2022	306,676.9547	3,394,204.184	823,279.137	1,014,699.592	20.958624
2023	301,750.3801	3,370,645.381	855,013.3554	1,007,022.151	9.276469
2024	310,225.7812	3,344,944.364	862,671.2312	997,318.2408	5.579007

Global Moran's I was calculated for each year using ArcGIS 10.8.1 (Table 5).

**Table 5:** Moran's I and spatial distribution types of traffic accidents in China, 2016–2024.

Year	Moran's I	z-score	*p*-value	Distribution type
2016	−0.0194	0.095	0.924	Random
2017	−0.418	−0.101	0.919	Random
2018	0.089	1.02	0.310	Random
2019	0.089	1.02	0.330	Random
2020	0.033	0.545	0.586	Random
2021	0.045	0.66	0.510	Random
2022	0.095	1.097	0.273	Random
2023	−0.002	0.26	0.794	Random
2024	0.023	0.501	0.616	Random

From 2016 to 2024, Global Moran's I fluctuated around zero, ranging from −0.418 to 0.095. The z-scores were small and all *p*-values exceeded 0. 05, so no significant global spatial autocorrelation was detected. Provinces with high and low accident totals were spatially interspersed rather than forming contiguous high- or low-incidence regions. Together, the concentration index and Global Moran's I characterize the national distribution as interprovincially uneven but weakly adjacent. Thus, provincial inequality did not translate into a continuous national cluster.

**Figure 3:**
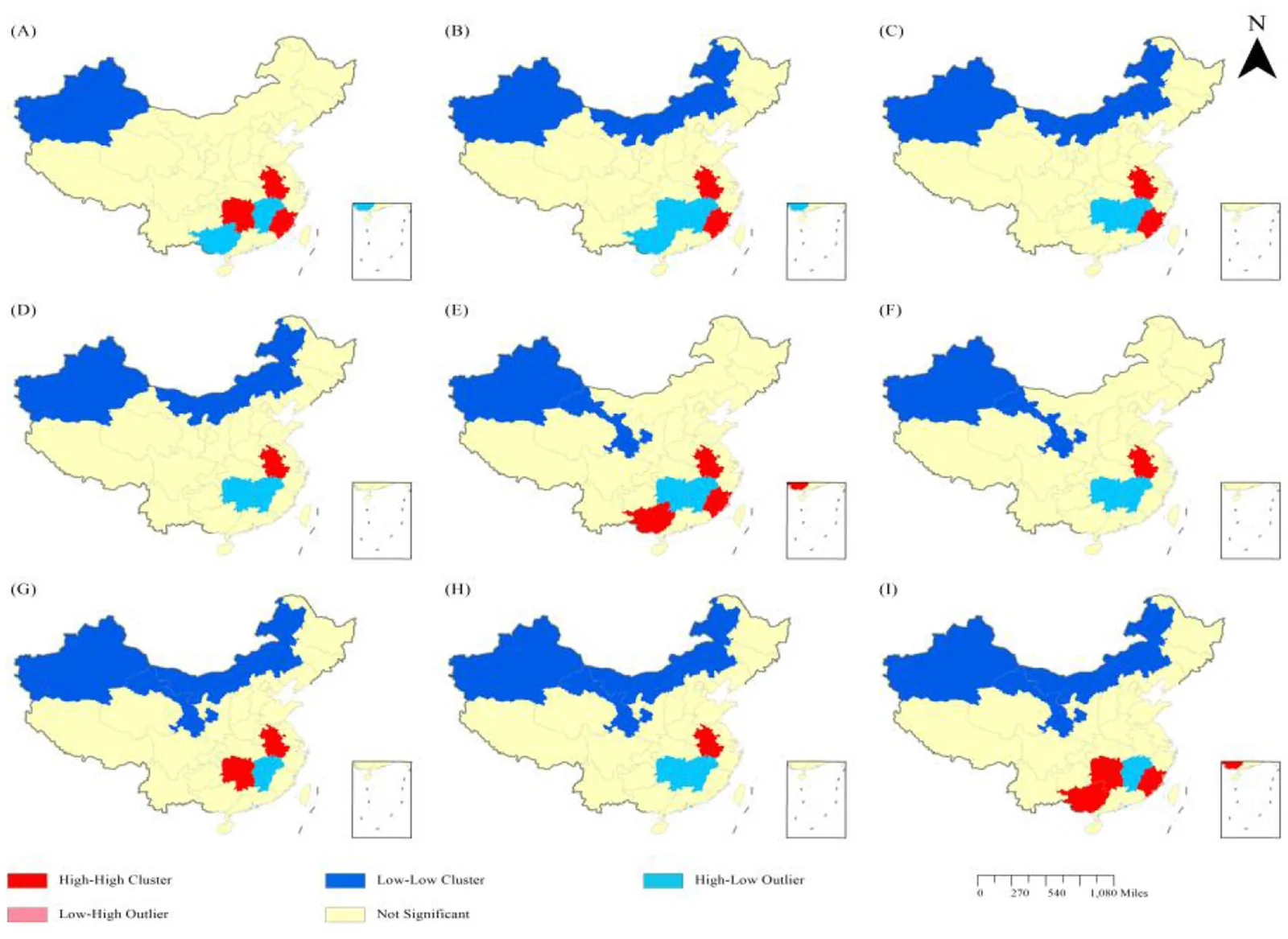
Local Moran's I maps of traffic accident distribution in China, 2016–2024. **(A–I)** correspond to 2016–2024, respectively.

**Figure 4:**
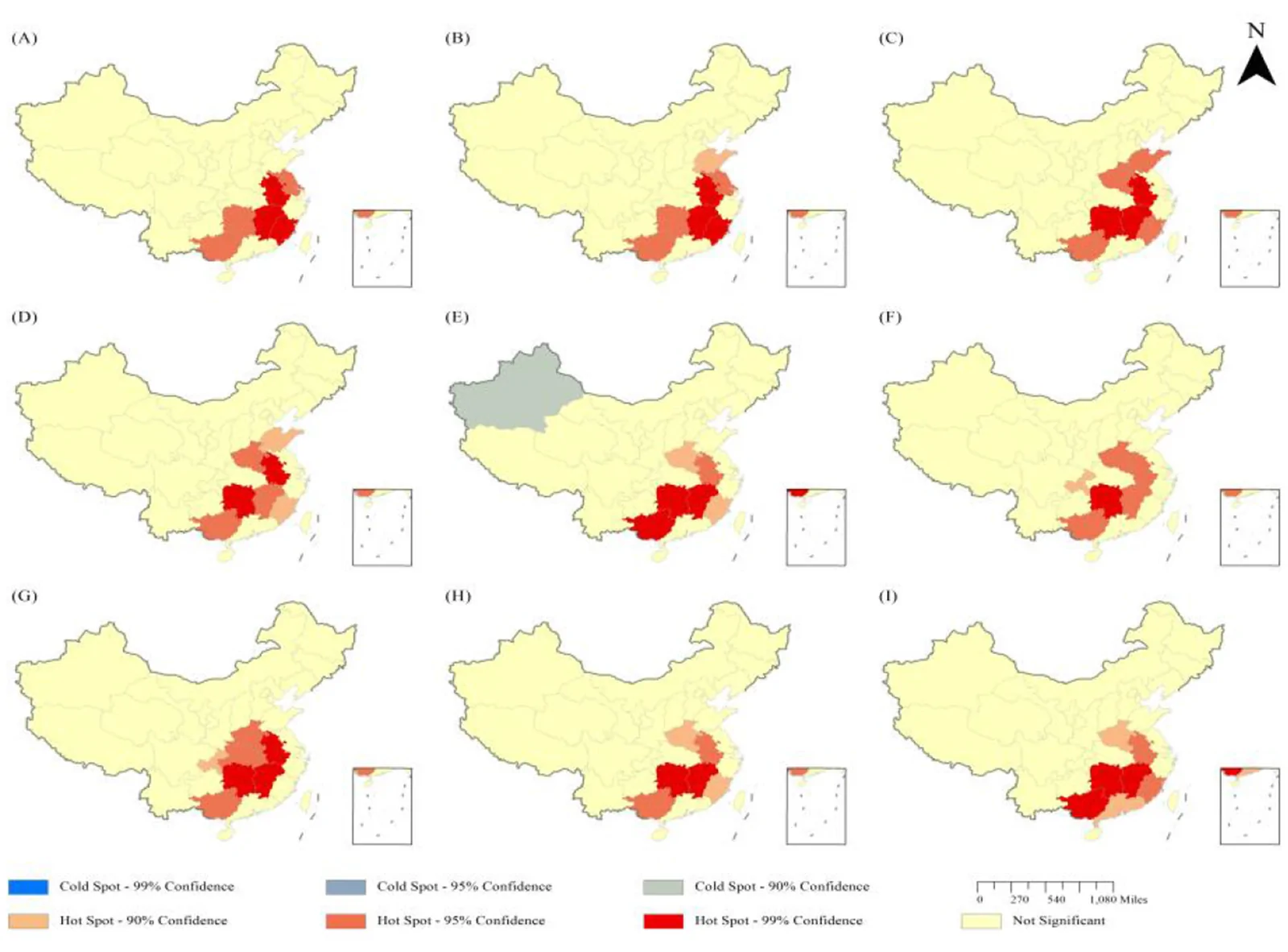
Hot spot and cold spot maps of traffic accident distribution in China, 2016–2024. **(A–I)** correspond to 2016–2024, respectively.

Local statistics nevertheless identified recurrent clusters. High-value clusters and hot spots were concentrated mainly in Guangxi, Anhui, Hunan, Jiangxi, and surrounding regions, with a “stable core and limited marginal” change. Anhui, Hunan, and Jiangxi repeatedly reached high confidence levels, while Henan, Fujian, Shandong, Hubei, Chongqing, and Guangdong entered the hot-spot set in particular years. Low-low clusters were persistent in Xinjiang, Inner Mongolia, Gansu, and nearby regions. Xinjiang was identified as a cold spot at the 90% confidence level only in 2020, while no statistically significant cold spots were detected in other years. Localized associations therefore persisted even though global autocorrelation was not significant (Table 6, Figures 3, 4).

**Table 6:** Hot spot provinces of traffic accidents in China, 2016–2024.

Year	90% confidence	95% confidence	99% confidence
2016	–	Jiangsu, Hunan, Guangxi	Anhui, Jiangxi, Fujian
2017	Shandong	Jiangsu, Hunan, Guangxi	Anhui, Jiangxi, Fujian
2018	–	Shangdong, Henan, Fujian, Guangxi	Jiangxi, Hunan, Anhui
2019	Shandong, Fujian	Henan, Jiangxi, Guangxi	Hunan, Anhui
2020	Henan, Fujian	Anhui	Guangxi, Hunan, Jiangxi
2021	Chongqing	Guangxi, Jiangxi, Anhui, Henan	Hunan
2022	Chongqing	Guangxi, Hubei, Henan	Jiangxi, Hunan, Anhui
2023	Henan, Fujian	Guangxi, Anhui	Jiangxi, Hunan
2024	Guangdong, Henan	Fujian, Anhui	Guangxi, Hunan, Jiangxi

### 4.2 Geodetector results

The factor detector shows that all ten indicators are significant at *p* < 0.05. Except for, all variables Eight have *p*-values below 0.001; per capita disposable income and educational attainment have *p*-values of 0.002 and 0.023, respectively (Table 7).

**Table 7:** Factor detector results for traffic accident distribution in China, 2016–2024.

Indicator	*q* statistic	*p*-value
Total road mileage (*X*_1_)	0.251	< 0.001
Urbanization rate (*X*_2_)	0.112	< 0.001
Gross regional product (*X*_3_)	0.388	< 0.001
Number of motor vehicle drivers (*X*_4_)	0.431	< 0.001
Number of civilian vehicles (*X*_5_)	0.369	< 0.001
Per capita disposable income (*X*_6_	0.074	0.002
Resident population density (*X*_7_)	0.411	< 0.001
Precipitation (*X*_8_)	0.227	< 0.001
Temperature (*X*_9_)	0.282	< 0.001
Educational attainment (*X*_10_)	0.041	0.023

The strongest individual factors are the number of motor vehicle drivers (*q* = 0.431), resident population density (0.411), gross regional product (0.388), and civilian vehicle ownership (0.369). Together, they represent the scale of traffic participation, population concentration, economic activity and motorization. Temperature (0.282), total road mileage (0.251), and precipitation (0.227) form a second tier, each with q-values above 0.20. Urbanization (0.112), disposable income (0.074), and educational attainment (0.041) have lower individual explanatory power but may still contribute through nonlinear interaction pathways.

**Figure 5:**
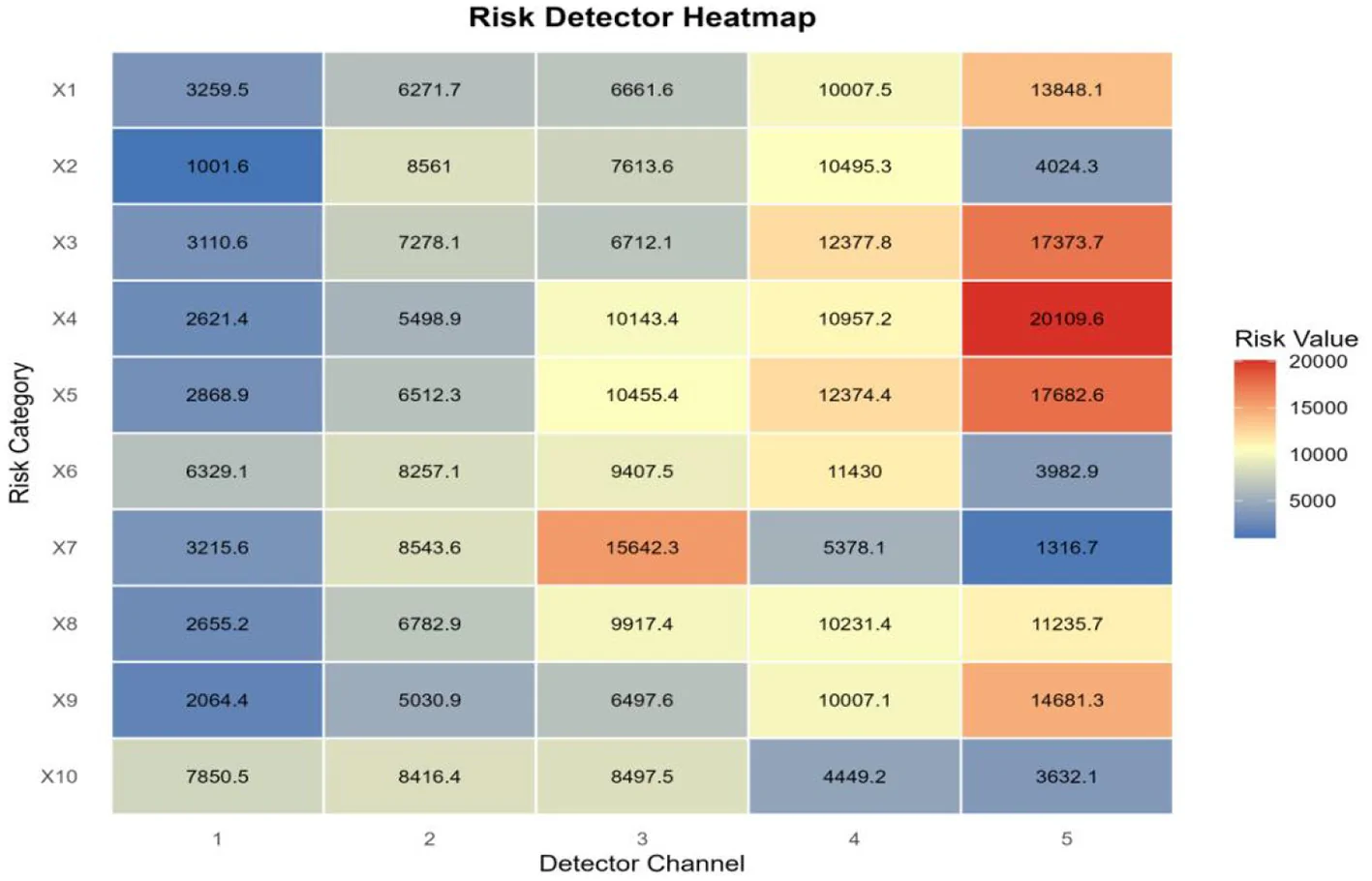
Risk detector results for traffic accident distribution in China, 2016–2024.

Risk-detector profiles differ across indicator. Mean accident counts increase across strata of road mileage, gross regional product, motor vehicle drivers, civilian vehicle ownership, precipitation, and temperature, although the spacing between strata varies. Urbanization and disposable income show nonlinear patterns, while resident population density follows inverted U-shaped pattern. For educational attainment, mean accident counts in the fourth and fifth strata are lower than those in the first three strata. The risk detector therefore adds direction and shape to the q-statistic ranking by distinguishing monotonic, threshold-like, and nonlinear profiles. Higher educational strata are associated with lower mean accident counts, although these provincial associations should not be interpreted as individual-level causal effects (Figure 5).

**Figure 6:**
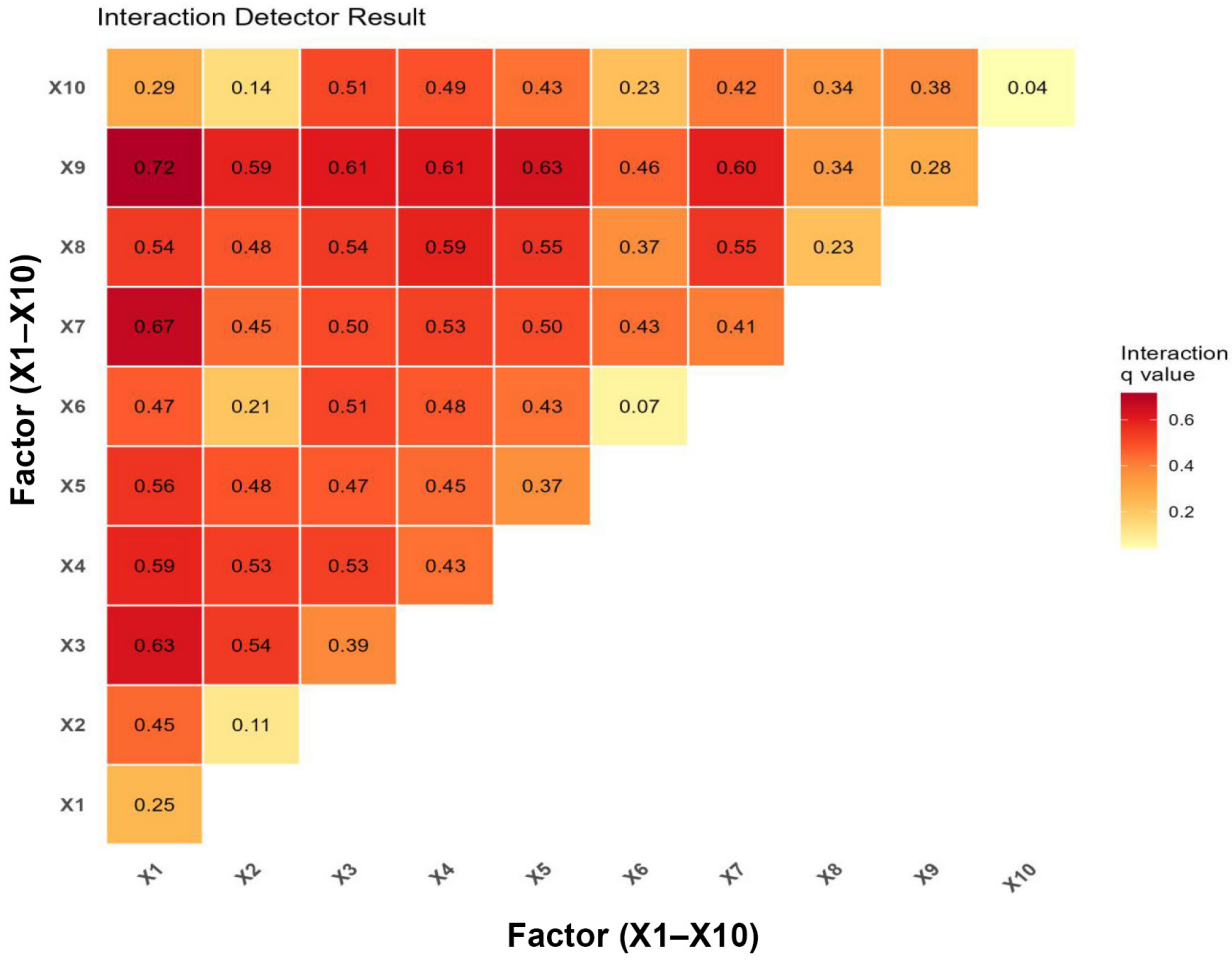
Interaction detector results for traffic accident distribution in China, 2016–2024. Note: X1 = total road mileage; X2 = urbanization rate; X3 = gross regional product; X4 = number of motor vehicle drivers; X5 = number of civilian vehicles; X6 = per capita disposable income; X7 = resident population density; X8 = precipitation; X9 = temperature; X10 = educational attainment. Interaction *q* values are dimensionless.

The interaction detector shows that every paired q value exceeds the corresponding individual effects. Bivariate enhancement and nonlinear enhancement are the dominant types, indicating that spatial heterogeneity is more effectively explained by interacting conditions than by isolated indicators (Figure 6).

Natural-environment interactions are among the strongest. Total road mileage and temperature (*X*_1_ ∩ *X*_9_) produce the highest q value (0.716) and show nonlinear enhancement, even though both variables have only moderate individual explanatory power. Temperature also interacts strongly with civilian vehicle ownership (*X*_5_ ∩ *X*_9_; *q* = 0.630) and resident population density (*X*_7_ ∩ *X*_9_), both showing bivariate enhancement. Precipitation produces nonlinear enhancement with total road mileage (*X*_1_ ∩ *X*_8_; *q* = 0.543) and urbanization (*X*_2_ ∩*X*_8_; *q* = 0.481). These combinations show that environmental conditions become more informative when considered together with infrastructure, motorization, and population exposure.

Infrastructure-socioeconomic interactions are strong. Total road mileage has moderate individual explanatory power, but its paired q values with demographic and socioeconomic indicators are substantially higher. Total road mileage and resident population density (*X*_1_ ∩ *X*_7_) reach *q* = 0.670, the second-highest interaction, while total road mileage and gross regional product (*X*_1_ ∩ *X*_3_) reach 0.630. Total road mileage and motor vehicle drivers (*X*_1_ ∩ *X*_4_) reach 0.590. The first two combinations show nonlinear enhancement and the third bivariate enhancement, indicating that infrastructure is most informative when considered together with the scale and intensity of activity.

## 5 Discussion

This study uses provincial panel data and spatial statistical methods to interpret the observed patterns of traffic accidents in China. These methods describe spatial patterns and statistical associations rather than establish causal relationships; the following explanations are therefore contextual interpretations supported by previous research.

### 5.1 Spatiotemporal distribution patterns

The results show an overall random distribution at the national scale but recurrent local hot and cold-spot clustering. These findings are not contradictory: the global statistic tests for a continuous national pattern, whereas local statistics identify particular neighboring configurations. The local pattern may be associated with regional differences in transport infrastructure, traffic exposure, and geographical conditions. Guangxi, Anhui, Hunan, and Jiangxi often appear in central or southern transport configurations with substantial through traffic. Their relatively high accident frequency may reflect greater exposure, although freight and heavy-vehicle movements were not measured directly (17, 36). Mountainous and hilly conditions in Guangxi and Hunan, including curves and downhill road, may further contribute to localized risk (5, 37). The evidence therefore supports province-specific monitoring rather than a simple east–west classification.

The SDE indicates expansion followed by contraction, and a general southwestward shift in the accident center. Between 2016 and 2020, transport infrastructure expanded rapidly in central and western China under national development strategies. In some areas, however, infrastructure growth may have outpaced traffic-management improvement, particularly where complex terrain increased operating difficulty. This conditions may partly contextualize the southwestward movement and early expansion of the ellipse (15, 26, 38). Since 2020, distribution has gradually contracted, and the north-south and east-west axes have not changed synchronously. The center movement should therefore be interpreted together with the changing size and orientation of the ellipse. These changes remain descriptive and should not be treated as evidence of the effects of particular strategy, policy, or external event.

### 5.2 Multidimensional driving mechanisms of spatial heterogeneity

The factor detector indicates that provincial variation is primarily associated with traffic exposure, while socioeconomic and environmental factors modify this relationship. The strongest factors are motor vehicle drivers (*X*_4_), resident population density (*X*_7_), gross regional product (*X*_3_), and civilian vehicle ownership (*X*_5_). More drivers and vehicles represent greater potential exposure, and economic activity generates passenger and freight demand (14, 39, 40). Gross regional product also reflects production and commercial exchange, but higher development may simultaneously support safer roads, enforcement, and emergency response. Civilian vehicle ownership represents potential rather than actual vehicle-kilometers traveled, so the resulting accident burden still depends on road capacity, vehicle mix, speed and management. These indicators are therefore related but not interchangeable. Population density also shows an inverted U-shaped profile rather than a simple linear effect. Intermediate-density provinces may combine strong travel demand, rapid motorization, and mixed urban–rural traffic without the lower exposure of sparse regions or the lower speeds, public transport, and refined management of the densest areas. Its safety implications therefore depend on travel intensity, road hierarchy, speed environment, and street design (41, 42).

Road infrastructure and natural conditions provide the external context in which traffic exposure operates. Total road mileage marks network extent but does not directly measure traffic demand, road class, quality, or operating conditions. A longer network may improve accessibility while increasing the number and diversity of potential conflict locations. Temperature and precipitation affect safety through pavement friction, visibility, driver fatigue, tires, and vehicle performance (43). Rainfall may increase hydroplaning and reduces visual judgement (2), whereas temperature fluctuations can influence both drivers and mechanical components. This effects may vary according to local infrastructure, traffic intensity, maintenance, and adaptation rather than acting uniformly across provinces (44).

Urbanization disposable income and educational attainment have weaker individual explanatory power but informative nonlinear profiles. Urbanization and income may increase mobility, commuting and vehicle ownership while also improving public transport, road quality, enforcement, vehicle safety, and emergency care. Their net association with accidents may therefore change across development stages (8, 27). Educational attainment is the only indicator whose highest strata correspond to lower mean accident counts. This association may reflect stronger knowledge of traffic rules, risk recognition, and compliance (24, 45). However, the provincial indicator cannot identify individual driver behavior, and its lower q value should not be interpreted as policy irrelevance.

### 5.3 Synergistic amplification of multiple risk factors

The interaction analysis shows that traffic accidents are associated with the coupled conditions of people, vehicles, roads, and the environment rather than any single factor. Natural factors have moderate individual explanatory power but form some of the strongest paired combinations. The interactions of road mileage and temperature (*X*_1_∩*X*_9_), the largest *q* value, may reflect more complex operating conditions on extensive networks under adverse weather (30).

Temperature also interacts strongly with vehicle ownership and population density, suggesting that weather-related vulnerability becomes more pronounced where motorization and population exposure are high (2, 29). The interaction between urbanization and precipitation further highlights the combined importance of rainfall, dense movements, intersections, drainage, and complex urban road environments (46). This patterns support weather-responsive warning, speed management, and maintenance measures, but they do not demonstrate that one factor causally modifies another.

Infrastructure-socioeconomic interactions reinforce this interpretation. Road mileage has moderate individual explanatory power but becomes substantially more informative when combined with gross regional product, motor vehicle drivers, or population density. Extensive road networks accommodate passenger and freight activity while the number and spatial distribution of drivers determine exposure across those network (17, 40). The road-mileage-GDP interaction reflects the convergence of economic activity and transport supply, and the road-mileage–driver interaction reflects the distribution of active traffic participation across the network. The strong road-mileage-population-density interaction shows that the safety implication of network scale depend on the intensity and spatial concentration of travel demand. The policy implication is not to restrict the network but to match it with access control, safe junctions, speed management, enforcement, and emergency capacity.

Collectively, the findings extend previous work by showing that provincial traffic-accident patterns are associated with combined conditions of transport infrastructure, socioeconomic development, and population exposure, and the natural environment. The nationwide longitudinal perspective complements city- and road-level studies, while the interaction detector shows why single-factor rankings provide an incomplete account of provincial heterogeneity. Region-specific strategies should therefore integrate infrastructure safety, exposure management, environmental risk mitigation, and coordination across recurrent local clusters. Table 8 summarizes the principal findings and implications.

**Table 8:** Summary of the main findings, interpretations, and public health implications.

Major finding	Interpretation	Public health implication
Global randomness with local clustering	Traffic risks are spatially heterogeneous	Region-specific prevention strategies
Southwestward shift of accident center	Relative Provincial Contributions changed over time	Improve road safety governance in western China
Traffic exposure variables dominate	Exposure–related variables explain most provincial variation	Coordinate exposure management and safe transport planning
Strong nonlinear interactions	Paired factors show enhanced joint explanatory power	Develop coordinated people–vehicles–roads–environment interventions

## 6 Conclusion

This study examined the spatiotemporal evolution and multidimensional driving mechanisms of provincial traffic accidents in China from 2016 to 2024. Accidents totals did not form significant continuous clusters at the national scale but showed global randomness, recurrent local clustering, and a general southwestward shift in the center of gravity. The ellipse expanded and then contracted, indicating changes in both the extent and orientation of provincial contributions over time. Motor vehicle drivers, resident population density, gross regional product, and civilian vehicle ownership have the greatest individual explanatory power. Temperature, precipitation, and total road mileage have moderate individual explanatory power but form strong interactions with exposure socioeconomic variables. The strongest combinations link road mileage with temperature, population density, and gross regional product. These results indicate that provincial accident heterogeneity is associated with the combined conditions of people, vehicles, roads, and the environment rather than a single determinant.

Traffic-safety governance should therefore move beyond uniform accident response and adopt differentiated prevention strategies. Provinces with persistently high accident totals should prioritize infrastructure improvements, targeted enforcement, exposure management and road-safety education. Regions with complex terrain or frequent adverse weather should strengthen road maintenance, meteorological warning systems speed management, drainage, and emergency response. At the national level, coordination among transport planning, regional development, and public health policy can help ensure that network expansion and increasing mobility are accompanied by appropriate safety capacity.

The provincial scale masks within-province variation and does not permit causal inference. Future studies could use city-, county-, or road-level data and incorporate traffic flow, freight transport, road design, speed, mobility, crash severity, and enforcement indicators. Sensitivity analyses using alternative spatial weights and discretization schemes would further assess the robustness of the geographical detector results.

## Data Availability

Publicly available datasets were analyzed in this study. Socioeconomic and transportation-related data were obtained from the National Bureau of Statistics of China, meteorological data were collected from the National Tibetan Plateau Data Center, and spatial administrative boundary data were sourced from the National Geomatics Center of China. The processed datasets used for analysis are available from the corresponding author upon reasonable request.

## References

[1] World Health Organization (2023). Global status report on road safety 2023. World Health Organization. Available online at: https://iris.who.int/server/api/core/bitstreams/46275f9f-ef66-4892-8ddd-a496ef8c1b74/content (Accessed May 25, 2026).

[2] Zhan Z-Y Yu Y-M Chen T-T Xu L-J Ou C-Q. Effects of hourly precipitation and temperature on road traffic casualties in Shenzhen, China (2010-2016): a time-stratified case-crossover study. Sci. Total Environ. (2020) 720:137482. doi: 10.1016/j.scitotenv.2020.137482 32145618

[3] James SL Lucchesi LR Bisignano C Castle CD Dingels ZV Fox JT . Morbidity and mortality from road injuries: Results from the Global Burden of Disease Study 2017. Inj Prev. (2020) 26:i46–i56. doi: 10.1136/injuryprev-2019-043302 31915274 PMC7571357

[4] Du W Fan H Li H Hao C. Trends in road traffic injury mortality and age-period-cohort analysis among children and adolescents in China and globally. Traffic Inj Prev. (2026) 27:462–71. doi: 10.1080/15389588.2025.2528147 40680209

[5] Jayasinghe BC Withanage NC Mishra PK. Evaluating geographical variations of road traffic accidents in Matara, Sri Lanka: a geospatial perspective to policy decisions. Rev Int Geomat. (2025) 34:707–29. doi: 10.32604/rig.2025.067395

[6] Le KG Tran QH Do VM. Urban traffic accident features investigation to improve urban transportation infrastructure sustainability by integrating GIS and data mining techniques. Sustainability. (2024) 16:107. doi: 10.3390/su16010107

[7] Hazaymeh K Almagbile A Alomari AH. Spatiotemporal analysis of traffic accidents hotspots based on geospatial techniques. ISPRS Int J Geo Inf. (2022) 11:260. doi: 10.3390/ijgi11040260

[8] Sun L-L Liu S Lan T Zou X-P. A study of the impact of traffic investment on traffic fatalities in China, 2004–2020. Chin J Traumatol. (2024) 27:380–8. doi: 10.1016/j.cjtee.2024.07.001 39299816 PMC11624423

[9] Wang H Liang G. Association rules between urban road traffic accidents and violations considering temporal and spatial constraints: A case study of Beijing. Sustainability. (2025) 17:1680. doi: 10.3390/su17041680

[10] Zhou N Zeng H Xie R Yang T Kong J Song Z . Analysis of road traffic accidents and casualties associated with electric bikes and bicycles in Guangzhou, China: A retrospective descriptive analysis. Heliyon. (2024) 10:e29961. doi: 10.1016/j.heliyon.2024.e29961 38694049 PMC11058882

[11] Amini RE Katrakazas C Antoniou C. Negotiation and decision-making for a pedestrian roadway crossing: A literature review. Sustainability. (2019) 11:6713. doi: 10.3390/su11236713

[12] Gorzelańczyk P Sokolovskij E. Using neural networks to forecast the amount of traffic accidents in Poland and Lithuania. Sustainability. (2025) 17:1846. doi: 10.3390/su17051846

[13] Gorzelańczyk P. The impact of road infrastructure quality and network density on road accident numbers in poland. Acta Tecnologí*a*. (2026) 12:17–21. doi: 10.22306/atec.v12i1.303

[14] Luo H Luo P Meng L. A robust geographically optimal zones-based heterogeneity model for analyzing the spatial determinants of national traffic accidents. GISci Remote Sens. (2025) 62:2448283. doi: 10.1080/15481603.2024.2448283

[15] Wang F Wang J Zhang X Gu D Yang Y Zhu H. Analysis of the causes of traffic accidents and identification of accident-prone points in long downhill tunnel of mountain expressways based on data mining. Sustainability. (2022) 14:8460. doi: 10.3390/su14148460

[16] Wang Y Zhai H Cao X Geng X. Cause analysis and accident classification of road traffic accidents based on complex networks. Appl Sci. (2023) 13:12963. doi: 10.3390/app132312963

[17] Gorzelańczyk P Sokolovskij E. Road accidents in the context of infrastructure and economic factors. Appl Sci. (2026) 16:2176. doi: 10.3390/app16052176

[18] Feng H Ning E Wang Q Li J. Cause analysis of traffic accidents in Ji'nan based on GIS. J Chongqing Jiaotong Univ. (2023) 42:124–31. doi: 10.3969/j.issn.1674-0696.2023.05.16

[19] Xiao D Ding H Sze NN Zheng N. Investigating built environment and traffic flow impact on crash frequency in urban road networks. Accid Anal Prev. (2024) 203:107561. doi: 10.1016/j.aap.2024.107561 38583284

[20] Kalantari M Zanganeh Shahraki S Yaghmaei B Ghezelbash S Ladaga G Salvati L. Unraveling urban form and collision risk: The spatial distribution of traffic accidents in Zanjan, Iran. Int J Environ Res Public Health. (2021) 18:4498. doi: 10.3390/ijerph18094498 33922679 PMC8122926

[21] Zhang G Lin J Li B. Risk factors for traffic violations and serious crash casualties in rural areas: a comparison of urban–rural differences in China. Front Public Health. (2026) 14:1754725. doi: 10.3389/fpubh.2026.1754725 41694537 PMC12894276

[22] Qu X Zhu X Xiao X Wu H Guo B Li D. Exploring the influences of point-of-interest on traffic crashes during weekdays and weekends via multi-scale geographically weighted regression. ISPRS Int J Geo Inf. (2021) 10:791. doi: 10.3390/ijgi10110791

[23] Salamati P. The level of education of drivers is a main human factor in road traffic crashes. Front Emerg Med. (2024) 8:e20. doi: 10.18502/fem.v8i2.15468

[24] Masoumi G Khorasani Zavareh D Haghdoust Z Ebadi A Moslehi S. Sociocultural factors affecting the incidence of road traffic crashes among drivers: An exploratory qualitative study. Arch Trauma Res. (2025) 14:91–9. doi: 10.48307/atr.2025.518101.1222

[25] Zhang K Wang S Song C Zhang S Liu X. Spatiotemporal heterogeneity analysis of provincial road traffic accidents and its influencing factors in China *Sustainability*. (2024) 16:7348. doi: 10.3390/su16177348

[26] Zhang Y Pan Z Zhu F Shi C Yang X. Quantitative estimation and analysis of spatiotemporal delay effects in expressway traffic accidents. ISPRS Int J Geo Inf. (2024) 13:407. doi: 10.3390/ijgi13110407

[27] Suphanchaimat R Sornsrivichai V Limwattananon S Thammawijaya P. Economic development and road traffic injuries and fatalities in Thailand: An application of spatial panel data analysis, 2012–2016. BMC Public Health. (2019) 19:1449. doi: 10.1186/s12889-019-7809-7 31684951 PMC6829991

[28] Cui P Abdel-Aty M Wang C Yang X Song D. Examining the impact of spatial inequality in socio-demographic and commute patterns on traffic crash rates: Insights from interpretable machine learning and spatial statistical models. Transp Policy. (2025) 167:225–45. doi: 10.1016/j.tranpol.2025.03.033

[29] Pourroostaei Ardakani S Liang X Mengistu KT So RS Wei X He B . Road car accident prediction using a machine-learning-enabled data analysis. Sustainability. (2023) 15:5939. doi: 10.3390/su15075939

[30] Xia H Liu R Zhou W Luo W. Modeling the causes of urban traffic crashes: Accounting for spatiotemporal instability in cities. Sustainability. (2024) 16:9102. doi: 10.3390/su16209102

[31] Wei X Cai J Liu C. Analysis of type and characteristic of spatial distribution of traditional villages in Jiangxi province. Mod Urban Res. (2017) 8:39–44. doi: 10.3969/j.issn.1009-6000.2017.08.007

[32] Zhang Y Jiang P Cui L Yang Y Ma Z Wang Y . Study on the spatial variation of China's territorial ecological space based on the standard deviation ellipse. Front Environ Sci. (2022) 10:982734. doi: 10.3389/fenvs.2022.982734

[33] Fischer MM Getis A. Handbook of applied spatial analysis: Software tools, methods and applications. Berlin: Springer (2010). doi: 10.1007/978-3-642-03647-7

[34] Anselin L. Local indicators of spatial association—LISA. Geogr Anal. (1995) 27:93–115. doi: 10.1111/j.1538-4632.1995.tb00338.x

[35] Wang J Li X Christakos G Liao Y Zhang T Gu X . Geographical detectors-based health risk assessment and its application in the neural tube defects study of the Heshun region, China. Int J Geogr Inf Sci. (2010) 24:107–127. doi: 10.1080/13658810802443457

[36] Wei X Tian S Dai Z Li P. Statistical analysis of major and extra serious traffic accidents on Chinese expressways from 2011 to 2021. Sustainability. (2022) 14:15776. doi: 10.3390/su142315776

[37] Yan X Zhu Z. City-level China traffic safety analysis via multi-output and clustering-based regression models. Sustainability. (2020) 12:3098. doi: 10.3390/su12083098

[38] Yang Y Jin L. Visualizing temporal and spatial distribution characteristics of traffic accidents in China. Sustainability. (2022) 14:13706. doi: 10.3390/su142113706

[39] Lord D Mannering F. The statistical analysis of crash-frequency data: A review and assessment of methodological alternatives. Transp Res A Policy Pract. (2010) 44:291–305. doi: 10.1016/j.tra.2010.02.001

[40] Elvik R. Some implications of an event-based definition of exposure to the risk of road accident. Accid Anal Prev. (2015) 76:15–24. doi: 10.1016/j.aap.2014.12.011 25560900

[41] Dumbaugh E Rae R. Safe urban form: Revisiting the relationship between community design and traffic safety. J Am Plann Assoc. (2009) 75:309–329. doi: 10.1080/01944360902950349

[42] Ewing R Dumbaugh E. The built environment and traffic safety: A review of empirical evidence. J Plann Lit. (2009) 23:347–367. doi: 10.1177/0885412209335553

[43] Theofilatos A Yannis G. A review of the effect of traffic and weather characteristics on road safety. Accid Anal Prev. (2014) 72:244–256. doi: 10.1016/j.aap.2014.06.017 25086442

[44] Zou Y Zhang Y Cheng K. Exploring the impact of climate and extreme weather on fatal traffic accidents. Sustainability. (2021) 13:390. doi: 10.3390/su13010390

[45] Mannering FL Bhat CR. Analytic methods in accident research: Methodological frontier and future directions. Anal Methods Accid Res. (2014) 1:1–22. doi: 10.1016/j.amar.2013.09.001

[46] Bilaşco Ş Man TC. GIS-based spatial analysis model for assessing impact and cumulative risk in road traffic accidents via analytic hierarchy process (AHP): Case study: Romania. Appl Sci. (2024) 14:2643. doi: 10.3390/app14062643

[47] Wang JF Xu CD. Geodetector: principle and prospective. Acta Geogr Sin. (2017) 72:116–34. doi: 10.11821/dlxb201701010

[48] Jenks GF. The data model concept in statistical mapping. Int Yearb Cartogr. (1967) 7:186–90.

